# Is It Really Over?

**DOI:** 10.1111/odi.15206

**Published:** 2025-07-25

**Authors:** Svyat Strokov, Nathalie Cardot‐Leccia, Hélène Raybaud, Sophie‐Myriam Dridi, Christine Voha

**Affiliations:** ^1^ Faculté de Chirurgie Dentaire Université Côte d'Azur, EA 7354 MICORALIS (Microbiologie Orale, Immunothérapie et Santé) Nice France; ^2^ Institut de Médecine Bucco‐Dentaire du CHU de Nice Nice France; ^3^ Laboratoire Central d'Anatomie et Cytologie Pathologiques (LCAP) Nice France

## 1 Case Report

**FIGURE 1:**
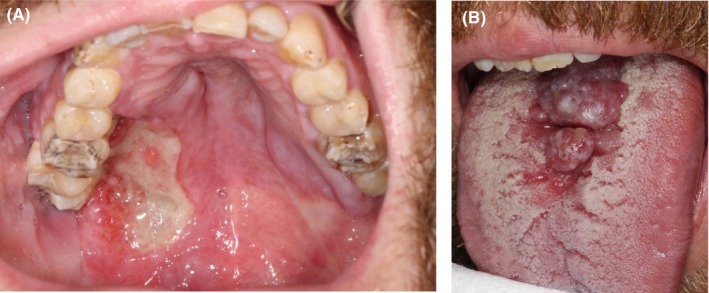
Clinical photography showing an extensive and deep ulcer occurring on the hard and soft palate (A), and a red multilobulated swelling occurring on the dorsal surface of the tongue (B).

A 32‐year‐old Eastern European man presents with a painless palatal ulcer and a red swelling on the tongue of 3 months' duration. He denies any significant medical history, avoids tobacco and drinks alcohol occasionally. He discloses a history of multiple homosexual encounters. Clinical examination reveals a large and deep ulcer on the right hard palate, extending into the soft palate. The adjacent teeth show marked mobility. Laboratory investigations revealed seropositivity for HIV‐1 (140 copies/mL), a CD4 T‐cell count of 464/mm^3^ and coinfection with hepatitis A virus, cytomegalovirus and syphilis. Incisional biopsy was performed and showed that both palatal and lingual lesions had similar histological features (Figure 1).

## 2 What is Your Diagnosis?

Based on the patient's history, physical examination and laboratory findings, which one of the following is the most suspicious diagnosis?

1. Necrotising sialometaplasia;
2. Squamous cell carcinoma;
3. Kaposi Sarcoma;
4. Histoplasmosis; and

## 3 Diagnosis

The final diagnosis was consistent with Kaposi's sarcoma involving the tongue and palate. The biopsy showed a tumour composed of spindle cells arranged in a fascicular architecture and expressing HHV‐8, ruling out other clinical differential diagnoses.

The duration of the ulceration (3 months) helps to exclude necrotising sialometaplasia. The absence of alcohol and tobacco intoxication and the young age of the patient do not support the diagnosis of squamous cell carcinoma. The ethnic origin of the patient and the absence of general symptoms (fever, fatigue, respiratory symptoms, etc.) do not immediately suggest histoplasmosis. Finally, ulcers associated with primary syphilitic chancre are typically less deep and do not result in bone destruction or tooth mobility.

Kaposi's sarcoma is classified as a low‐grade angiosarcoma that is consistently associated with human herpesvirus 8 (HHV‐8) infection. There are four types of Kaposi's sarcoma, with two indolent subtypes: classical Kaposi's sarcoma, which affects Mediterranean populations, and endemic African Kaposi's sarcoma. The two aggressive forms are associated with immunosuppression, either iatrogenic (following organ transplantation or immunosuppressive treatment) or viral in origin (related to HIV infection) (Bishop and Lynch 2024). In the case presented, it is an epidemic form associated with HIV seropositivity. Its polymorphic clinical presentation may mimic many other diseases, as it may manifest as macules, plaques or violaceous nodules, as well as nonspecific ulcerations (Shetty 2006).

**FIGURE 2:**
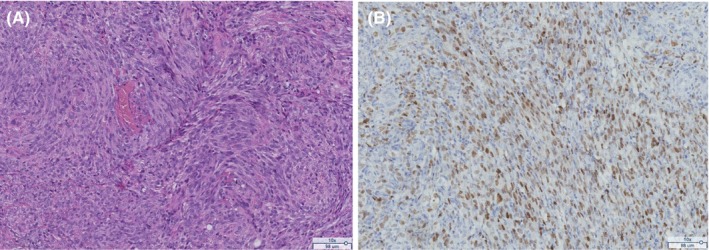
(A) Photomicrograph (HE, original magnification 100×) showing bundles of spindle cells dissecting vascular slit‐like spaces. (B) Photomicrograph (anti‐HHV‐8, original magnification 100×) showing a diffuse expression of HHV‐8 by the neoplastic spindle cells.

The most common histological finding is bundles of spindle cells dissecting vascular spaces. These cells consistently express HHV‐8, which is of great diagnostic value as some forms may be confused with simple pyogenic granulomas. Treatment consists primarily of starting antiretroviral therapy as early as possible. Additional treatments (surgery, radiotherapy or chemotherapy) may be considered to reduce local symptoms or in severe and disseminated forms (Thieringer et al. 2021). The overall outcome is influenced by treatment, viral load, CD4 cell count, CD4/CD8 ratio and nadir CD4 cell count (Bishop and Lynch 2024). Despite the significant decline in AIDS‐related Kaposi's sarcoma following the introduction of antiretroviral therapy in 1996, Kaposi's sarcoma remains the most common cancer associated with HIV (Yarchoan and Uldrick 2018; Guedes et al. 2021). Dental practitioners must remain cautious and consider Kaposi sarcoma as a possible differential diagnosis, including non‐nodular ulcerated lesions (Figure 2).

## 4 Outcome

The patient is managed in a multidisciplinary approach in consultation with an infectious disease specialist, and antiretroviral treatment consisting of lopinavir–ritonavir and lamivudine is initiated. After 3 months of follow‐up, he remains alive with a significant reduction in oral ulceration.

## Author Contributions

**Svyat Strokov:** formal analysis, investigation, conceptualization, writing – review and editing. **Nathalie Cardot‐Leccia:** validation, writing – review and editing. **Hélène Raybaud:** methodology, validation, visualization. **Sophie‐Myriam Dridi:** methodology, validation, writing – review and editing. **Christine Voha:** conceptualization, validation, methodology.

## Consent

The patient reported in this manuscript provided written informed consent for the publication of the case details.

## Conflicts of Interest

The authors declare no conflicts of interest.
